# High-resolution biologging of an Atlantic bluefin tuna captured and eaten by a supposed orca

**DOI:** 10.1038/s41598-024-80744-0

**Published:** 2024-11-26

**Authors:** Jessica L. Rudd, Ghalia Abel, Francisco Baringo, Samantha Birch, Barbara A. Block, Martin A. Collins, Renaud de Stephanis, Owen M. Exeter, Francesco Garzon, Christophe Guinet, Thomas W. Horton, David Righton, Jeroen van der Kooij, Matthew J. Witt, Serena Wright, Lucy A. Hawkes

**Affiliations:** 1 Hatherly Laboratories, University of Exeter, Prince of Wales Road, Exeter, EX4 4PS UK; 2 Conservation, Information and Research on Cetaceans (CIRCE), Cabeza de Manzaneda 3, 11390 Pelayo-Algeciras, Spain; 3 Centre for Environment, Fisheries and Aquaculture Science, Pakefield Road, Lowestoft, NR33 0HT UK; 4 Department of Oceans, Stanford University, Hopkins Marine Station, Pacific Grove, CA USA; 5 British Antarctic Survey, NERC, High Cross, Madingley Road, Cambridge, CB3 0ET UK; 6 Centre for Ecology and Conservation, University of Exeter, Penryn Campus, Penryn, Cornwall, TR10 9FE UK; 7 Centre d’Etudes Biologiques de Chizé, Centre National de la Recherche Scientifique, 79360 Villiers-en- Bois, France; 8 School of Environmental Sciences, University of East Anglia, Norwich, NR4 7TJ UK

**Keywords:** Performance, Hunting, Predation, Biologging, Ecology, Behavioural ecology, Ecosystem ecology

## Abstract

Biologging has been used on a range of wild animals to document spectacular feats of migration and behaviour. We describe the pursuit, capture, and ingestion of an adult Atlantic bluefin tuna (*Thunnus thynnus*) (175 cm, estimated weight: 81 kg), which was instrumented with a biologging tag, by a predator, most likely an orca (*Orcinus orca*). The predation event lasted over 19 min, with the tuna exhibiting elevated activity (max acceleration 3.12 g) and a rapid ascent from 126 m at 3.6 m.s^− 1^ followed by death and handling at the surface. Orca were separately recorded using video tags, capturing and handling tuna cooperatively in a manner consistent with the tuna data. We then present the longest orca accelerometry dataset from the ingested MiniPAT tag, with diel patterns of activity and 77 feeding events. These unique datasets provide insight into the energetic dynamics of two of the ocean’s fastest predators.

## Introduction

Predation plays a crucial role in structuring ecosystems through changing the abundance, distribution and behaviour of both predator and prey^1^. Tracking technologies can provide novel insight into predator-prey interactions and food web dynamics which would otherwise be challenging to obtain through conventional methods^2,3^. Tag sensors such as pressure, light levels and accelerometry can be used to reveal when, where and how animals perform in a predator-prey system. Variation in depth data may help narrow down possible predators^4^ while changes in temperature data may reflect whether a tagged individual has been ingested by an endothermic rather than an ectothermic species^2,5^ and shed light onto the predator’s physiology^4^ or feeding rates^6,7^. Acceleration data can inform on handling times as well as specific kinematic signatures of the predators foraging behaviour^8,9^. Designing tagging studies to investigate predation can be challenging and introduce a number of biases^3^, however much of the knowledge to date has been gained opportunistically through unintended natural mortality of tracked individuals. We present data documenting the predation of an Atlantic bluefin tuna (hereafter tuna) carrying a biologging tag by a presumed orca in the Bay of Biscay (approximate location 45°39’00” N 9°22’52.5” W). The tuna was tracked with a MiniPAT tag (Wildlife Computers, WA, USA) recording depth, temperature, tri-axial acceleration and light level (at 0.2 Hz resolution) and was recovered by a member of the public, giving access to the data archive at native resolution.

## Results and discussion

### Atlantic bluefin tuna behaviour and predation event

**Fig. 1:**
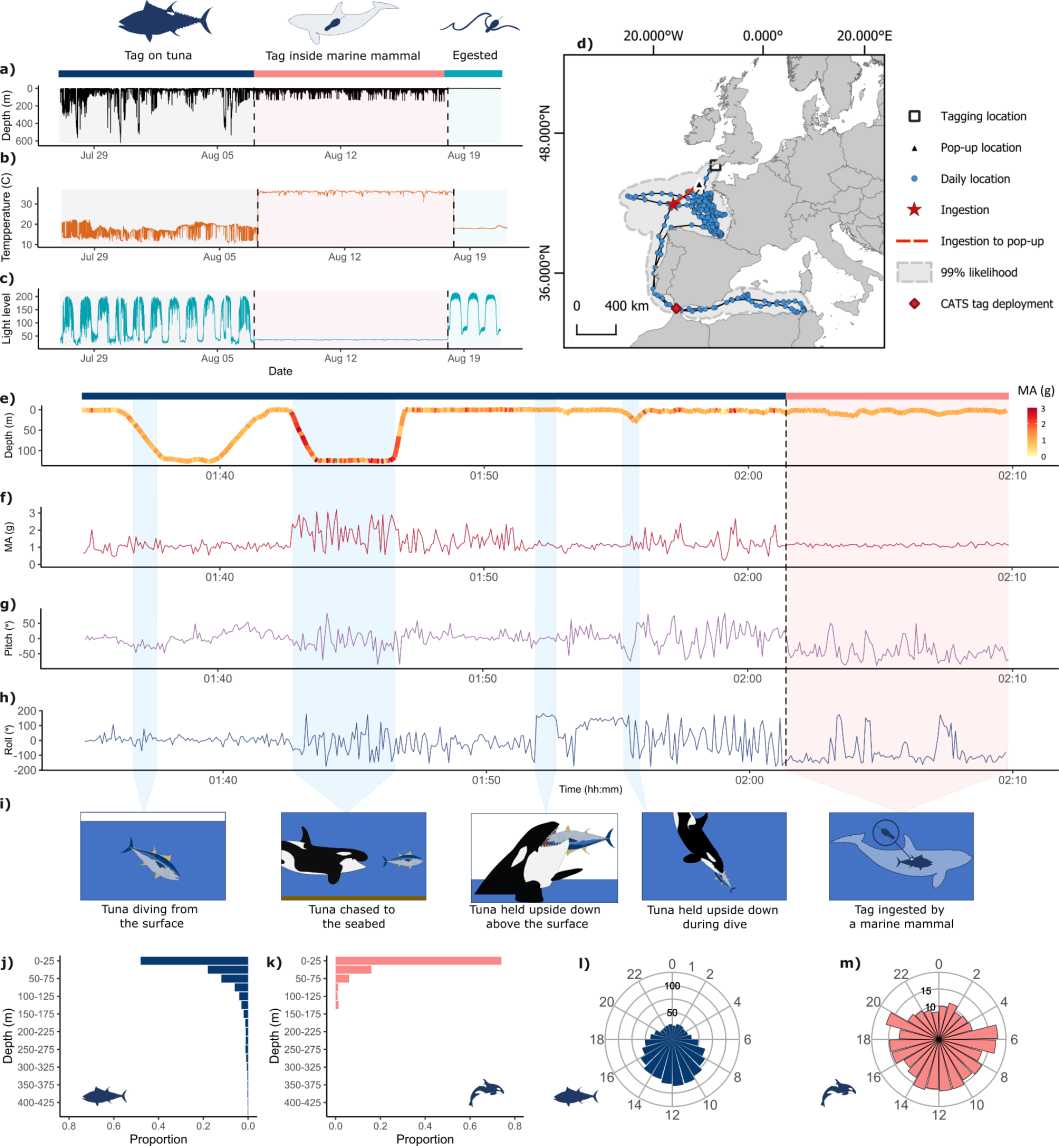
Predation of an Atlantic bluefin tuna by a marine mammal. Time series of the depth (**A**), temperature (**B**) and light levels (**C**) recorded by the tagged tuna for the 11 days leading up to the predation event, shaded in grey. The 11 days the tag was ingested by the marine mammal are shaded in pink, and the three days following expulsion of the tag are shaded in light blue with corresponding pink and blue horizontal bars above the plot. The predation event is marked by sudden increase in the temperature above 30 °C, light levels close to zero and changes in depth patterns. Diel patterns of light as well as ambient temperature returned following expulsion of the tag, which is seen floating at the surface in the depth trace. (**D**) Best daily locations of the tuna during its 289 days at liberty, tracked from the southwest of England, into the Mediterranean until predation (red star). Position of the marine mammal was interpolated between the transmitted location and pop-up location after tag expulsion (red dashed line). (**E–H**) Time series leading up to the predation event marked by the vertical dotted line with the horizontal blue segment representing the period the tag was attached to the tuna, and pink segments to when the tag was ingested. The depth profile (**E**) is coloured by the magnitude of acceleration (MA, g), time series of the magnitude of acceleration (g) (**F**), as well as pitch (°) and roll (°) (**G**, **H**). Shaded sections correspond to events depicted in the graphical representations in (**I**). (**J**) represents the proportion of time the tuna (dark blue) and the marine mammal (pink) (**K**) spent at different depths during their tracking duration. The mean hourly depths for both of these periods are represented by circular barplots for the tuna (**L**) and marine mammal (**M**) respectively.

After release from the southwest coast of England, the tuna migrated via the Bay of Biscay into the Mediterranean before returning, travelling a minimum straight-line distance of 16,045 km over 289 days (Fig. 1D). On the 7th August 2019 (approximate location 45°39’00” N 9°22’52.5” W off the continental shelf) between 01:02 and 01:42 UTC, the tuna made repeated trips from the surface to 91–126 m depth. A sudden burst in activity associated with a dive from the surface to 126 m depth at a vertical velocity of 1.1 m.s^− 1^ was then recorded, with a magnitude of acceleration increasing to 150% (max acceleration 3.12 g) that of the whole deployment (Fig. 1E-H). Depth was maintained for ~ 3 min before the tuna surged to the surface at a vertical velocity of 3.5 m.s^− 1^ for 35 s, a rate 4.4 times greater than dives in the preceding hour. The tag recorded erratic and highly fluctuating changes in pitch (-85 to 84°) and roll (-178 to 180°) as the tuna swam to the surface before measuring two prolonged upturned periods (for 45 and 90 s with a mean roll angle of 160° ± 11° and 134° ± 19° respectively; Fig. 1H-I). The tuna may have been captured and killed at this point, with the tag then recording surface manipulation by the predator. A sudden spike in light levels, lasting 45 s, was recorded during the first upturned interval (Supplementary Fig. 1), suggesting the tag, which was likely still attached to the tuna at one point of attachment, may have been held above the sea surface during the half-moon. Light levels were nearly twice as high as the mean light intensity recorded during astronomical twilight for the five proceeding days and similar to levels recorded during the onset of dawn and dusk. Both upturned phases were broken by short dives (to 11 and 30 m respectively) before the tuna was presumably taken back to the surface. A second highly active phase followed at the surface (mean magnitude of acceleration: 1.17 ± 0.47 s.d. g for 6.7 min), associated with extreme fluctuation in pitch and roll as the tag recorded the movement of tuna presumably being consumed and/or the tag anchoring system becoming loose compared to usual swimming patterns (Supplementary Fig. 2). Then at 02:01 UTC, 19.2 min after the initial burst in activity, the temperature abruptly increased from 18.4 to 32.4 °C within 20 s, as the tag (and presumably the tuna) was ingested. Surges in acceleration, postural data and depth profile leading to the ingestion event suggest the tuna was predated upon at liberty rather than caught by longline or recreationally and then predated upon. The predator’s behaviour and internal temperature were then recorded opportunistically for a further 11 days before the tag was regurgitated and drifted ashore.

### Predator identification

Large bluefin tuna (> 1.5 m) have few known predators^10–13^, however changes in depth profiles to surface orientated diving and internal temperatures averaging 35.9 °C ± 0.5 °C suggest the tuna was predated upon by a marine mammal rather than a regionally endothermic shark such as white sharks (*Carcharodon carcharias)* which are known to feed on bluefin tuna^10^ but have considerably lower stomach temperature ranging 23.4–27.4 °C^14,15^. Large (> 2.4 m) tagged bluefin tuna have also been reported predated by unidentified marine mammals off the cost of Morrocco^16^. Three predators were then deemed potential candidates based on prevalence in the Bay of Biscay, dive behaviour, diet and internal body temperature: the orca or killer whale (*Orcinus orca*), the false killer whale (*Pseudorca crassidens*) and the pygmy killer whale (*Feresa attenuata*) (Table 1).

**Table 1:** Qualitative prevalence, diet, dive behaviour and body temperature of candidate predators.

Species	Prevalence in French & Spanish waters	Record of Atlantic bluefin tuna in diet	Can dive to 156 m?	Mean dive duration (min)	Max dive duration > 11.1 min	Diel behaviour	Internal temperature (C°)
Pygmy killer whale	Yes - rare^17^	Unknown	Yes 368 m^34^	4.2–4.7^34^	No^34^	No^34^	35.9 ^29^
False killer whale	Yes - rare^18,19^	Yes^12^	Yes 1330 m^35^	2.15 ^35^	Yes 14.6 ^35^	Unknown	36-37.2^33^
Orca	Yes vagrant^11,18,20,23^	Yes ^11,24,26,28^	Yes 221 m^40^ 254 m^36^ 300 m^37^ 379 m^9^ 480 m^38^ 767.5 m^42^ 1087 m^72^	2.1-2.8^41^ 3.41^42^ 3.5^40^ 3.9-4.1^38^	Yes 15.9^42^	Yes + Nocturnal feeding^36,41,42,49,73^	35.3-36.3^30^ 35.9-36.3^31^36^32^ 37.1–38^33^

While both false killer whales and pygmy killer whales are sporadically sighted in the Bay of Biscay^17,18^, both species are typically distributed in warm temperate and tropical waters^19^ and neither are known to regularly prey on Atlantic bluefin tuna^12^. Comparatively, North Atlantic orca sightings are more commonly reported within the Bay of Biscay^18,20,21^, peaking between April-September^11,21,22^ during which the habitat is highly suitable for orca distribution, with the extent expanding along the Iberian Peninsula continental shelf into the Bay of Biscay and Eastern Atlantic between July-September^21^. Bathymetry was found to be the most important predictor for orca presence, exploiting deeper waters in the summer compared to other seasons^21^, coinciding with the migration and suitable foraging habitat of Atlantic bluefin tuna^23–25^. Orca not only directly hunt tuna^26,27^, but also depredate fisheries targeting tuna on longlines^23,27,28^. The ingested tag recorded an overall internal body temperature of 35.9 °C ± 0.5 °C (range 31–36.8 °C) which is similar to rectal temperature reported in a captive pygmy killer whale (35.9 °C^29^), as well as stomach and rectal temperature ranges recorded in captive orcas (35.3–36.3 °C^30^; 35.9–36.3 °C^31^; 36 °C^32^). False killer whales reported warmer mean temperatures of 36.6 °C (range 36–37.2 °C^33^).

All three predators are known to dive deeper than the maximum dive depth of 156 m recorded in the present study (Table 1). Maximum dive depths of 368 m have been reported in pygmy killer whales, however they were found to dive deeper at night than during the day compared to the marine mammal in the present study, and the maximum dive duration was shorter at 9 min^34^ compared to 11.1 min. False killer whales can dive up 1330 m and have a maximum dive duration of 14.6 min^35^. Like the marine mammal in the present study, deeper depths were reported during the day than at night, however mean dive depth (41 ± 92 m) and dive duration (2.15 ± 1.88 min) were longer for false killer whales^35^. Depth patterns in the present study were most similar to those reported in orca. Dive behaviour can vary between study sites and are influenced by environmental conditions experienced by the animals, so comparison between dive behaviour in the present study and previous work may not match exactly. For example, across their range orcas have been found to dive as deep as 254 m in transient killer whales^36^, 300 m in Northern Resident killer whales^37^, 379 m in Southern Resident killer whales^9^, 480 m in Eastern North Pacific Offshore killer whales^38^ and as deep as 1083 m in the Southern Ocean^39^ (Table 1). Maximum depths of 221 m were recorded for North Atlantic orca tracked in northern Norway^40^, with a mean range of 9–10 m (data from four individuals), compared to an average depth of 13 m recorded in our data. The maximum dive duration of 11.1 min reported in current study falls within previously reported maxima for different orca populations ranging between 10 and 15.9 min^36,37,41,42^, while the average dive duration is similar to durations recorded in both adult and juvenile orcas across a range of behaviours^43^. It seems most likely that an orca captured, manipulated, and eventually ate the tuna and tag, and from hereon we will therefore, with caveats, refer to the predator as a supposed orca. These data are first ever to reveal the frequency of feeding in wild orca in the Bay of Biscay, and the longest orca accelerometry data to date (next longest dataset 96.3 h^40^) albeit at very low sampling frequency (every 5 s vs. > 50 Hz) and not externally attached compared previous multi-sensor tags tracking studies.

Orcas have been previously described using an endurance-exhaustion technique to target small bluefin tuna (< 1.5 m), chasing prey beyond their aerobic limits for up to 30 min at speeds of 6 m.s^− 126^. For larger tuna, such as the fish in the present study, orcas are expected to switch to cooperative hunting strategies. It took 19 min for the tagged tuna to be successfully captured and the tag consumed. Although tuna could simply retreat to deeper depth to evade predation^44,45^, following the initial dive, the tuna maintained a depth around 126 m for 3 min before being chased to the surface.

While the light geolocation model suggests that the bathymetry at the last known tuna tracking location was > 4000 m, the maximum depth of the tuna was only 126 m, suggesting either a degree of error of the last location or that the maximum depth was behaviourally driven. Light geolocation models can have spatial errors of 100s of kilometres and generate more accurate estimates when supplied with greater volumes of high-quality data^46^. In the two days preceding the predation event, the observation scores assessing the quality of location estimates by Wildlife Computers’ GPE3 model were relatively low (mean: 12.3 ± 21, where a score of 100 corresponds to very high accuracy), indicating high uncertainty in the tuna’s location and underlying bathymetry. This suggests that the tuna may have been in shallower waters than predicted by the light-based geolocation model, particularly off the Iberian Peninsula and Bay of Biscay continental shelf region where depth gradients change abruptly over short distances. Orcas have been observed herding prey against natural or artificial obstacles^26^ and can have hunting strategies where some individuals lie ahead in ambush while others chase prey to the surface^47^. However, the 90% likelihood of the light geolocation estimate for the last known location still falls over 3000 m deep (Supplementary Fig. 3), suggesting that bathymetry may not be limiting the tuna’s dive depth. Temperatures experienced by the tuna prior to predation event remained above the minimum temperature recorded across the full deployment (minimum: 9.8 °C), suggesting temperatures were not limiting dive depth. The tuna may then have been subjected to cooperative herding - flanked by multiple orcas and chased to the surface where it was captured, flipped and held above the water as suggested by the light and postural data. Based on the frequency of feeding events on the day of capture, other tuna in the area may also have been herded and caught. Prey harassment by orcas has been commonly reported^39,48^, with observations of several prey species held at the surface or thrown in the air^26,39,47^ including harbour porpoises^39^ of similar size to the tagged tuna^39^.

### Dive behaviour following tag ingestion

Following ingestion of the tag, swimming behaviour changed markedly from the tuna’s mean depth of 50 ± 94 m to the marine mammal’s 12.9 m ± 21.8 m. Despite the tuna’s deep diving capacity (max depth: 1,211 m), 81% of its tracking duration was spent within the marine mammal’s depth range (0–156 m). Both the tuna and marine mammal displayed diel patterns of depth use, swimming deeper during the day than at night (tuna day depth: 73 ± 76 m, range 0-1211 vs. night: 31 ± 45 m, range 0–1128 m, Wilcoxon rank sum test W = 56172, *p* < 0.001; marine mammal day depth: 15 m ± 24 m, range: 0-156 vs. night: 10 m ± 19 m, range: 0–149 m, W = 4507235, *p* < 0.001; Fig. 1L-M). The marine mammal dives lasted on average only 80 ± 85 s, with the longest dive lasting 11.1 min. The marine mammal dives that were greater than 20 m depth (mean ± s.d.= 50.3 ± 29.4 m) lasted on average 3.6 min and were longer during the day (86.5 ± 94.3 s) than at night (72.6 ± 72 s; Wilcoxon rank sum test, W = 6,408,193, *p* = 0.02). Diurnal diving behaviour has been recorded in several orca populations^36,41,42^ including North Atlantic orcas tracked off Norway in response to light conditions and the diel vertical migration of prey^40,49^. Patterns of diel vertical migration have been reported for bluefin tuna tracked elsewhere within the North Atlantic Shelf Region^50^ .

### Feeding behaviour

**Fig. 2:**
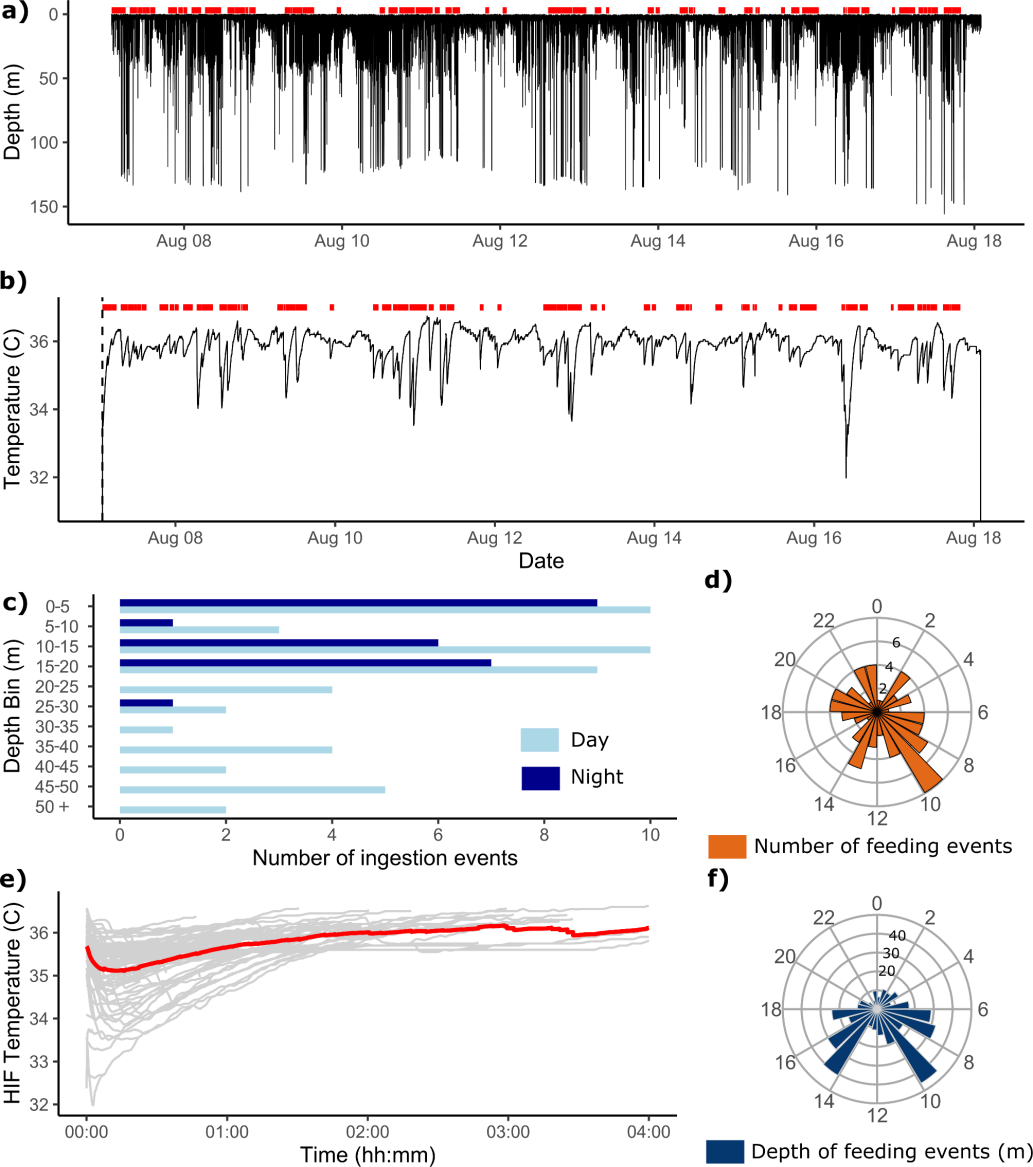
Marine mammal feeding behaviour. (**A**) Depth profile and (**B**) internal body temperature recorded for 11 days the tag was ingested by the marine mammal. Red horizontal bars represent the duration of individual heat increment of feeding events. (**C**) Number of ingestions by depth bin and by time of day, where light blue bars represent ingestions occurring during daylight hours, and dark blue bars those at night. (**D**) Circular boxplot of the total number of ingestions by hour of the day, with the most occurring at 09:00. (**E**) Temperature profile of each heat increment of feeding (HIF) event (*n* = 77, light grey), with the overall mean temperature profile of ingestion events shown in red. (**F**) Circular boxplot of the mean depth (m) of ingestion events by time of day.

Knowledge of food passage times for Cetacea is limited, and most estimates originate from animals in managed care^51^. For example, it took 56 h for a captive orca to egest a temperature sensor pill^33^. This suggests that the biologging tag probably became lodged somewhere in the marine mammal’s digestive tract over the 11-day period, before being regurgitated as temperature fluctuation resulting from cold prey ingestion was recorded throughout the tracking duration without the expected temperature attenuation if the tag had travelled further through the orca’s digestive track to defecation. The tag recorded periodic sharp drops in temperature dissociated from the depth profile, which were likely a result of cold-water ingress during feeding, rather than drinking, as dietary free water is the main source of water obtained by cetaceans^52^. These temperature profiles align with patterns of feeding events recorded in captive and wild marine mammals equipped with stomach temperature pills^6,7,33,53–55^, implying probable ingestions by the supposed orca. At least 77 such feeding events were recorded over the 11-day period (an estimated 6.9 ingestions a day ± 2.8, range 4–13; Fig. 2A). On nine occasions, multiple temperature anomalies associated with a sudden drop in internal body temperature followed by several smaller temperature drops were recorded within a 30-minute period, indicative of multiple feeding events (Fig. 2B, Supplementary Fig. 4). The greatest number of ingestions was recorded on 7th August, the day the tagged tuna was eaten. Ingestion events occurred throughout the 24-hour period, with over a quarter of the events (*n* = 23) occurring between 07:00 and 11:00 AM UTC (Fig. 2D). The time intervals between feeding events ranged from 1.3 min to 13.8 h, averaging 3.6 h (± 3.4 h s.d.) and were similar between night and day (Welch two sampled t-test, t=-0.36, *p* = 0.72; day: 3.3 h, night 3.6 h). The marine mammal fed at significantly deeper depths during the day (23 ± 26 m, range: 0.5–120 m) than at night (8.2 m ± 5.9 m, W = 872, *p* < 0.01, Fig. 2C), during which feeding only occurred within 21 m of the surface (Fig. 2F), including the predation event of the tagged tuna where both the initial chase and subsequent ingestion of the tag occurred within 1 m of the surface. Nocturnal feeding behaviour has also been reported from acoustic data in North Atlantic orca populations tracked in Iceland^56^. Counter-colour shading of orcas serves as effective camouflage from prey during daylight hours^57^, with 66% of ingestions (33 out of *n* = 50) occurring during the bottom and ascent phases of dives during daylight hours compared to at night.

Feeding events could be visualised when the tag transiently recorded temperatures higher than the overall mean as a result of chemical and physical processes associated with digestion, or dropped dramatically following the start of a new feeding event (see Methods). Following each meal, the marine mammal’s internal body temperature increased on average by 1.1 ± 0.9 °C, reaching as much as 36.6 °C, and lasted on average for 1.6 h (± 1.1 s.d., range 6 min to 5 h, Fig. 2E). The time between consecutive meals increased significantly with peak temperatures following ingestions (excluding multi-feeding events for which internal temperature did not surpass the average body temperature, suggesting digestion was incomplete between events; R^2^ = 0.25, *P* < 0.001, y = 35.9 + 0.12x). The heat increment of feeding (HIF) is the heat produced by specific dynamic action and is the metabolic heat produced during digestion^58^. A scarcity of values exists for the HIF of marine mammals, particularly cetaceans^51^. HIF depends on the size and composition of the meal, as well as the age and nutritional state of the animal in question^59^. Quantifying the duration of HIF is only possible for captive animals for which resting temperature, meal size, composition and fasting can be controlled^51^. HIF has been suggested to increase with meal size^6,60,61^, so the supposed orca may have delayed feeding following larger meals associated with greater peak temperatures. This has been demonstrated in free ranging tagged grey seals (*Halichoerus grypus*), for which the time between meals increased with the size of the previous meal^53^. Although feeding rates were lower in the tracked marine mammal than reported in other fish-feeding orca populations, different foraging behaviours have been reported between sympatric populations and between sexes^9,62^. Orcas that eat tuna may then obtain larger proportion of ingested energy from fewer, larger animals than numerous smaller meals as observed in herring-feeding orca in Norway which would require an estimated 85–578 herring/day to sustain a ~ 4,000 kg male orca^40^.

## Diving and predation behaviour of a tagged orca

**Fig. 3:**
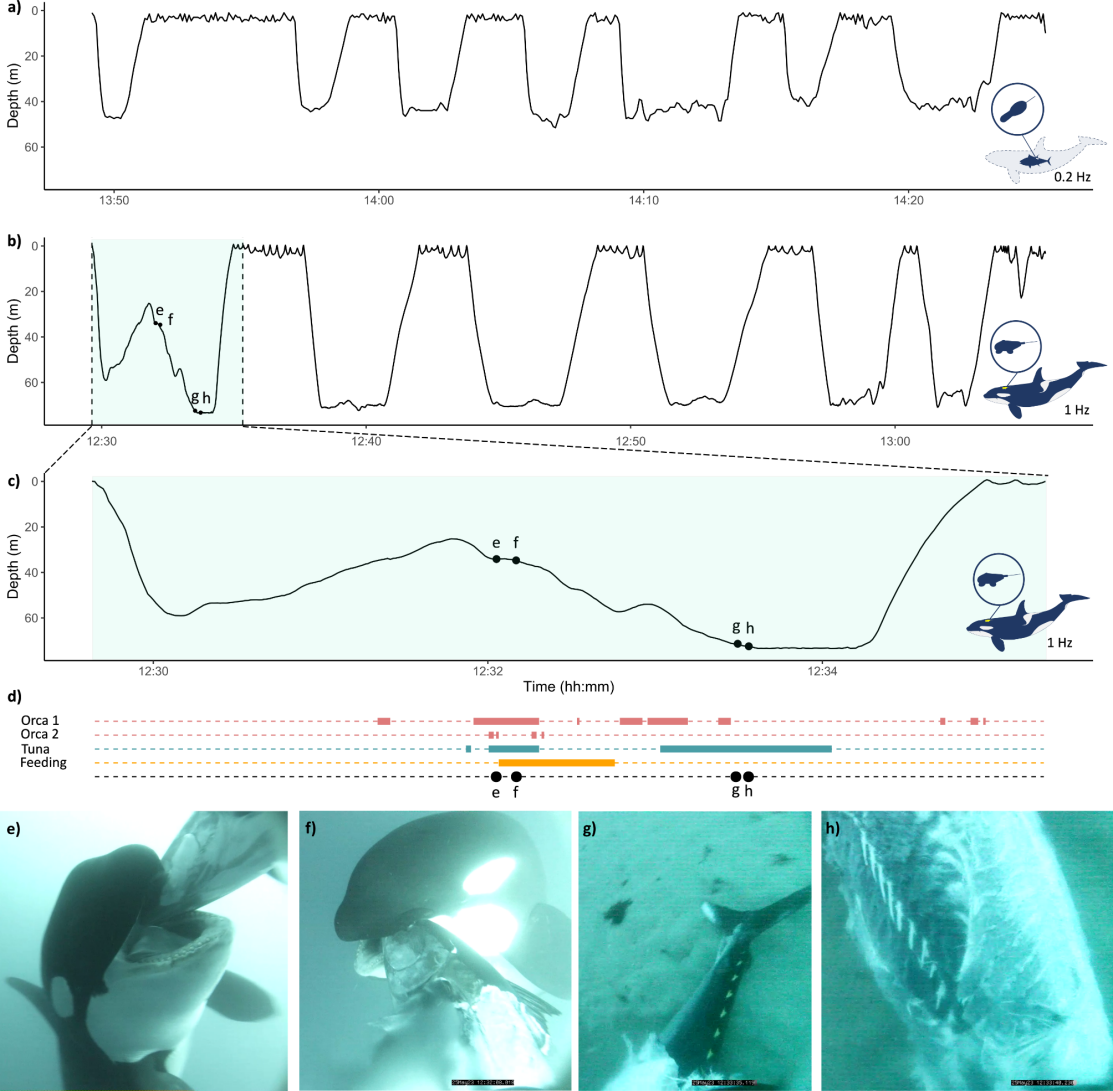
Predation event of a bluefin tuna by orcas captured on video. (**A**) Example of a 36-minute depth profile recorded by the ingested MiniPAT tag (0.2 Hz) by a supposed orca. (**B**) Depth profile of the full 36-minute deployment of the orca tracked with the CATS Cam measured at 1 Hz, where the first dive associated with a bluefin tuna feeding event is highlighted in light green. (**C**) Inset of the first dive made by the tracked orca, where letters represent the timing of the associated events depicted in (**E–H**). (**D**) Timeline of events recorded during the first dive of the tracked orca. Pink segments depict the presence of additional orcas in view of the camera, turquoise when the tuna was in view, and orange when the orca was being eaten by the tagged orca, defined by audio data. Black dots correspond to the timing of associated screenshots of the feeding event (**E–H**).

An adult female orca was tagged off the Straights of Gibraltar in May 2023 with depth, temperature, tri-axial acceleration and video data collected for 36.05 min before the tag fell off. Dive patterns were similar to those recorded by the ingested tuna tag (Fig. 3A-B). The tagged orca made repeated dives to the seabed to an average maximum depth of 64.4 ± 18.3 m (range: 22.9–73.5 m) with a mean duration of 3.7 min ± 1.6 (range: 5.3–0.6 min). Between deep dives, the orca spent 84 ± 48 s at the surface (range:30–162 s), taking 3–12 breaths (mean 7 ± 3). Whilst at the surface, the orca made shallow dives to 3.4 ± 1.3 m (range 1.1–7.5 m) lasting on average 12.4 ± 4.9 s (range: 3–21 s). Overall, the tagged orca swam deeper than the marine mammal that ingested the tag (Wilcoxon rank sum test, W = 107429941, *p* < 0.001). However, the deployment was considerably shorter and not representative of the whole range of behaviours to be expected from an orca. When only comparing dives greater than 20 m deep, dive durations were similar (tagged orca: 3.7 ± 1.6 min; ingested tag: 3.6 ± 1.6 min; Wilcoxon rank sum test, W = 3734.5, *p* = 0.42; Fig. 3A-B), while the average maximum depth remained significantly deeper for the tagged orca (mean: 64.4 ± 18.3 m) than the ingested tag 50.3 ± 29.4 m (Wilcoxon rank sum test, W = 2357, *p* = 0.03).

Video data revealed that within 2 min of the tag deployment, fragments of fish were recorded floating past the animal-borne camera, after which a large bluefin tuna was observed at 25.3 m depth, followed by a second orca swimming from the depth (Fig. 3C-D). The second orca captured the tuna at 27 m, holding its head between its jaws and exposing the tuna’s ventral side as a third orca came into view holding onto the fish’s abdomen. The third orca tore part of the abdomen and musculature along the tuna’s flank, revealing its internal organs, whilst the second orca maintained the tuna in position to help with tearing of the skin (Fig. 3E-F). The tagged orca then fed on the tuna’s organs, stripping the dorsal musculature whilst the second orca maintained the tuna steady in its jaws. Whilst feeding, the tagged orca drifted from 34 m to 57.3 m, with audio of chewing behaviour recorded for 47 s. The tuna then became out of site as the second orca fed, with the tagged orca following the carcass down to the seabed to 73 m deep. On the seabed, the tagged orca filmed a second orca feeding on the tuna carcass. By this point, most of the tuna had been consumed including the head, with mostly the end of caudal fin and rib cage remaining (Fig. 3G-H). The tagged orca inspected the carcass for 68 s, moving it around before deeming no meat was left and swam back to the surface. The tagged orca interacted with the tuna for a total of 91 s across the duration of the deployment (4% of the video). Conspecifics were in view of the camera on 11 occasions, for a total of 70 s (3.2% of the video), with a maximum of two additional orcas captured in the same shot. The longest interaction between conspecifics lasted 26 s, during which an orca presented the tagged orca with the bluefin tuna. The tuna carcass was traded between different orcas at least four times, highlighting prey sharing and group foraging behaviour.

While the tagged orca did not record the capture event of the bluefin tuna, the recording revealed cooperative feeding between orcas, with handling of the prey to facilitate tearing the meat from the fish. Type B Antarctic killer whales have been observed pulling Weddell seals in opposite directions to help strip the carcass^63^. Similar behaviour may have been recorded by the tagged tuna which was handled for prolonged periods prior to ingestion (Fig. 1). The video data also revealed that orcas fed first on the internal organs before consuming the dorsal musculature, which is where the tag was attached on the tagged tuna. This may be why intense fluctuations in pitch and roll were recorded by the MiniPAT as the tuna was being consumed and the tag became partially loose before the tag itself was ingested. The video data also highlighted how multiple individuals shared a single prey. Therefore, feeding events recorded by the ingested tag may represent portion sizes rather than consumption of whole prey. Prey sharing is often the norm for orca populations feeding on large marine mammals, where all members of a group are involved in feeding^63^, whereas in fish-feeding Pacific resident killer whales, prey sharing was nonreciprocal and only directed towards close maternal kin^64^. Salmonids were shared regardless of prey size and torn apart for social feeding rather than to facilitate the consumption of large prey items^65^.

These serendipitous data have provided insight into one of the most dramatic predatory interactions in the oceans, documenting an event where maximum metabolic performance of air breathing and water breathing top marine predators is pitted against one another. These findings also shed insight into natural sources of mortality for large mature bluefin tuna which have few known predators in the Northeastern Atlantic Ocean and highlight the use of archival tags for future studies attempting to address predator-prey interactions^51^. Since several populations of orca depredate long-line fisheries^23,27,66–68^, there could be scope for baiting prey with archival tags as done for white sharks^8^ to answer basic physiological questions and validate estimates of energy intake for bioenergetic models.

## Methods

### Ethics declaration

All tuna work was carried out under licence from the UK Home Office (P23C6EFD2) and dispensation from the UK Marine Management Organisation. All tuna work was reviewed and approved by the University of Exeter’s Animal Welfare and Ethical Review Board (AWERB). Work was also carried out under the ICCAT Atlantic-Wide Research Programme for Bluefin Tuna (GBYP), which is funded by the European Union, several ICCAT CPCs, the ICCAT Secretariat, and other entities (see https://www.iccat.int/gbyp/en/overview.asp). All orca work was carried out under licence from the Ministerio para la Transicion Ecologica y el Reto Demographico (MITECO) of the Government of Spain (SGBTM/BDM/AUTSPP/32/2022) and approved by MITECO. All methods in the manuscript were performed in accordance with the relevant guidelines and regulations of the UK Home Office, AWERB and MITECO and are reported in accordance with ARRIVE guidelines.

### Bluefin tuna capture and tagging

On 2nd November 2018 a tuna measuring 175 cm (curved fork length) with an estimated weight of 81 kg was captured off the south coast of Cornwall, UK (45°39’00” N 9°22’52.5” W) as part of “Thunnus UK” (www.thunnusuk.org), a tracking study aiming to examine the increased presence of tuna in the region^69^. The tuna was tagged on deck with a MiniPAT tag (118 × 38 mm (length and width), Wildlife Computers, WA, USA) attached using two percutaneous darts next to the 2nd dorsal fin, and an additional loop to minimise excessive movement^50,70^. The tag recorded depth, temperature, tri-axial acceleration and light level at 0.2 Hz and was programmed to detach from the tuna after one year, or if the tag remained at a constant depth +/- 2.5 m for three days. Following tagging, the tuna was returned to the water and towed at the rear of the vessel at slow speeds (< 5 knots) for 2 min to aid reoxygenation using a BOGA grip (Pratiko, Italy).

### Data processing

All data processing and analysis was conducted in R. Accelerometry data were adjusted to account for tag attachment using the “tagtools” package in R. Body pitch and roll were extracted using additional “tagtools” functions. The magnitude of acceleration was calculated as a proxy for activity, as the square root of the sum of squares of each raw accelerometer axis:

1 $${\text{MA }} = \sqrt {Ax^{2} + Ay^{2} + Az^{2} }$$

### Spatial data

Tuna tracking locations were reconstructed from tag data using the Global Position Estimator 3 (GPE3, Wildlife Computers) following methods described in Horton et al.^71^. Minimum straight-line distance between daily locations were calculated using the function “geodDist” in the “oce” R package to measure the total minimum straight-line distance of the tracks.

### Characterisation of the predation event and inferring predators

The primary indication of the predation event by a marine mammal was the sudden increase in temperature above 30 °C and constant low light levels maintained throughout the ingestion period, along with surface-oriented dive behaviour. Timing of the expulsion of the tag was similarly quantified as the moment at which temperature dropped below 30 °C and the tag drifted back to the surface, with a return of diel light changes.

The most likely predators were deduced from the list of North Atlantic marine mammals^19^ and sightings data from the ORCA database (orca.org.uk) based on behavioural and physiological data retrieved from the ingested tag, along with published information on diet, internal body temperature, depth use and prevalence in the Bay of Biscay. Predation records of bluefin tuna were searched for each predator. Diving behaviour was examined for each candidate predator to see whether they can dive to the maximum depth recorded by the ingested tag (156 m) and whether their maximum dive duration is similar to the maximum dive duration recorded in the present study (11 min). Stomach/internal body temperature was also compared between candidate predators where the information was possible as well as whether feeding behaviour has been recorded at night.

### Dive behaviour

Dives in the present study were considered as swimming behaviour below 2 m that lasted more than 15 s (3 data points) and with a maximum depth greater than 4 m to account for the 0.5 m accuracy of the tag and the fact that it was ingested. Deep dives were characterised as dives greater than 20 m deep. Dive duration and maximum dive depth was retrieved for each dive. Surface intervals were defined as the amount of time the marine mammal spent shallower than 4 m between consecutive dives. Diel differences in dive depth and dive duration were compared using Wilcoxon rank sum tests for daytime and nighttime periods of each day between sunset and sunrise times estimated for the last day of tuna tracking data.

### Characterisation of feeding frequency

Since dietary free water is the main source of water obtained by cetaceans^52^, sudden drops in internal body temperature were considered associated with feeding events rather than drinking sea water. As a conservative measure, the start of a feeding event was identified by a sharp drop in internal temperature below 35.7 °C lasting more than 10 s associated with the ingestion of cold food, followed by a steep steady rise of the temperature. A feeding event was considered to have ended when (i) the internal temperature returned above the mean body temperature (35.9 °C) or (ii) changed in a manner indicative of the start of new feeding event characterised by a significant dip in internal temperature (Supplementary Fig. 1). For each ingestion event, the minimum and peak temperatures were recorded. Peak temperature was defined as either the maximum temperature recorded prior to a multi-feeding event or when the temperature became stable (± 0.1°) over a 3 min period. The time to reach the peak temperature as well as the time between the start of consecutive ingestion events (feeding interval) were also measured.

Linear regressions were used to test whether a relationship existed between peak temperature and the time to reach peak temperature, and between peak temperature and feeding interval length. Since several multi-feeding events were recorded, where several ingestions occurred within less than 30 min or internal temperature had not risen above 35.7 °C suggesting digestion had not ended, subsequent regressions were conducted on a subset of data for which the peak temperature was > 35.7 °C.

The depth of ingestion events was considered as the depth at which the internal temperature made an initial sharp dip. Mean depths of ingestion events were compared between night and day using Wilcoxon rank sum tests. To investigate whether there were any temporal differences, the number and mean depth of ingestion events as well the time interval between events were compared between hours of the day, daytime and nighttime periods as well as between consecutive days.

### Orca tag deployment

A single adult female orca was tagged on the 25th May 2023 off the western reaches of the Straights of Gibraltar (36°03’50.0"N 5°54’49.7"W) with a suction cup CATS biologging device (CATS tags, Customizable Animal Tracking Solutions, www.cats.is*)* equipped with a forward-facing camera (1280 × 720 pixels at 26.95 FPS), a time depth recorded, accelerometer, magnetometer, gyroscope (all at 100 Hz) and VHF telemetry (Model MM130B, Advanced Telemetry Systems, Minnesota, USA) to aid with tag retrieval. To deploy the tag, a small research vessel approached a focal group of orcas and waited for an individual to surface. The tag was mounted on a 2–3 m adjustable pole and temporarily attached with suction cups on the head of the orca as it surfaced. Following the tagging event, the pod left the vicinity of the boat. The tag was recovered three hours later once it had popped off 2.4 km from the tagging site (36°02’54.2"N, 5°53’40.9"W) and was recovered using a dip net after triangulation of the position using a R-5000 VHF receiver (Communications Specialist, Inc).

### CATS cam analysis

All data was processed and analysed in R. Depth data was temperature corrected using the R package “tagtools”. The “fix_pressure” function finds minima in dive profile that are consistent with surfacing and uses the depth at these points to fit a temperature regression. Depth data was down-sampled from 100 Hz to 1 Hz to remove noise in the data. Dives were defined as swimming behaviour deeper than 1 m, and classified into surface dives if the maximum depth of a dive was shallower than 10 m and deep dives if deeper than 20 m. Dive duration and maximum dive depth was retrieved for each dive. Since dives were predominantly down to the seabed, diving behaviour of deep dives were compared with dives greater than 20 m recorded by the ingested tag using a Wilcoxon rank sum test.

Video data were observed in VLC Media Player (version 3.0.3) at the native speed to ascertain information on the presence of conspecifics, presence of prey and timing and duration of feeding events from the audio component of the video. Feeding events from the audio data were characterised by distinctive ripping and chewing sounds. Corresponding depth was retrieved for the start of each encounter.

## Data Availability

The data supporting the findings in this study will be made available upon acceptance of the manuscript on Movebank and the Cefas Data Portal. Data requests should be made to the corresponding author.
